# Heat Stroke or Acute Coronary Syndrome? A Summer Collapse With an Unexpected Cardiac Twist

**DOI:** 10.7759/cureus.111529

**Published:** 2026-06-26

**Authors:** Muhammad Zulqarnain, Ilyas Mukhtar

**Affiliations:** 1 Acute Medicine, Whittington Hospital, London, GBR

**Keywords:** acute coronary syndrome, coronary artery disease, heat stroke, percutaneous coronary intervention, syncope, troponin

## Abstract

Heat stroke is a life-threatening medical emergency characterised by severe hyperthermia and central nervous system dysfunction. Cardiac manifestations, including troponin elevation and electrocardiographic abnormalities, are well recognised and may mimic acute coronary syndrome (ACS). Distinguishing heat-related myocardial injury from true ACS can therefore be challenging. We report the case of a 60-year-old man who presented after being found unresponsive in his garden during a hot summer day. His pre-hospital core temperature exceeded 42.2°C, and he had a markedly reduced level of consciousness. Following active cooling measures and supportive treatment, his neurological status improved rapidly. Initial investigations demonstrated elevated high-sensitivity troponin T levels without acute ischaemic electrocardiographic changes. Owing to his cardiovascular risk factors and unexplained collapse, he was treated for non-ST-elevation ACS. Transthoracic echocardiography subsequently demonstrated regional wall motion abnormalities, and coronary angiography revealed significant proximal left anterior descending artery disease requiring percutaneous coronary intervention. This case highlights the diagnostic challenge of differentiating heat stroke-related myocardial injury from concomitant coronary artery disease and emphasises the importance of careful cardiovascular assessment in patients presenting with heat stroke and elevated cardiac biomarkers.

## Introduction

Heat stroke is a medical emergency characterised by a core body temperature exceeding 40°C accompanied by central nervous system dysfunction. It represents the most severe form of heat-related illness and can result in multiorgan dysfunction affecting the neurological, renal, hepatic, and cardiovascular systems. Cardiac involvement in heat stroke is increasingly recognised and may manifest as electrocardiographic abnormalities, elevated cardiac biomarkers, myocardial dysfunction, arrhythmias, and heart failure [1,2].

The pathophysiology of heat stroke-induced myocardial injury is complex and likely multifactorial, involving direct thermal injury, systemic inflammatory responses, endothelial dysfunction, microvascular thrombosis, and impaired myocardial perfusion [2]. Consequently, patients with heat stroke may present with elevated troponin concentrations and electrocardiographic abnormalities that closely resemble acute coronary syndrome (ACS) despite the absence of significant obstructive coronary artery disease [2,3].

Conversely, exposure to extreme heat may itself precipitate acute coronary events. Heat stress increases cardiovascular workload through dehydration, haemoconcentration, tachycardia, and increased myocardial oxygen demand, potentially resulting in myocardial ischaemia in susceptible individuals with underlying coronary artery disease [4]. As global temperatures continue to rise, clinicians are likely to encounter increasing numbers of patients presenting with heat-related illness and associated cardiovascular complications.

Recent studies have further highlighted the association between heat stroke, myocardial injury biomarkers, and adverse cardiovascular outcomes. Elevated cardiac biomarkers, including troponin and natriuretic peptides, have been associated with disease severity and poorer prognosis in patients with heat stroke [5]. The Fourth Universal Definition of Myocardial Infarction (UDMI) defines myocardial injury as a cardiac troponin concentration above the 99th percentile upper reference limit (URL), with acute myocardial injury requiring a rise and/or fall in troponin values. In contrast, myocardial infarction (MI) requires evidence of acute myocardial injury in conjunction with clinical evidence of myocardial ischaemia. Type 1 MI results from acute atherothrombotic plaque disruption, whereas Type 2 MI arises from an imbalance between myocardial oxygen supply and demand in the absence of acute coronary thrombosis [6]. Distinguishing heat stroke-induced myocardial injury from concomitant ACS, therefore, remains challenging and has important implications for management and prognosis. We present a case of classic heat stroke associated with elevated troponin levels in which further investigation revealed significant coronary artery disease requiring percutaneous coronary intervention (PCI).

## Case presentation

A 60-year-old gentleman was brought to the emergency department on a hot summer day when the ambient temperature was approximately 33°C. At approximately 10:00 am, he was found unresponsive in the garden of his home by his daughter, who immediately called an ambulance. According to his daughter, he had been in the garden for approximately 30-60 minutes before being discovered.

On assessment by the ambulance crew, he was noted to be extremely hot to the touch, with a recorded core body temperature greater than 42.2°C. His blood pressure was 130/100 mmHg, heart rate 125 beats per minute, respiratory rate 22 breaths per minute, and oxygen saturation 95% on room air. His initial Glasgow Coma Scale (GCS) score was 6 (E1V3M2). Capillary blood glucose was 19.5 mmol/L.

Immediate cooling measures were initiated, including transfer into an air-conditioned ambulance, application of ice packs to the axillae and groins, and administration of cold intravenous fluids.

His medical history included insulin-dependent diabetes mellitus and hypertension. He had a 10-pack-year smoking history and consumed approximately 4-8 units of alcohol per week since adolescence. Socially, he lived with his daughter, mobilised with a walking stick, and was independent in most activities of daily living.

Upon arrival at the emergency department, his level of consciousness had improved considerably. His core temperature had decreased to 40.5°C, and his GCS had improved to 14 (E3V5M6). Active cooling measures were continued, and a variable-rate intravenous insulin infusion was commenced for hyperglycaemia.

**Figure 1:**
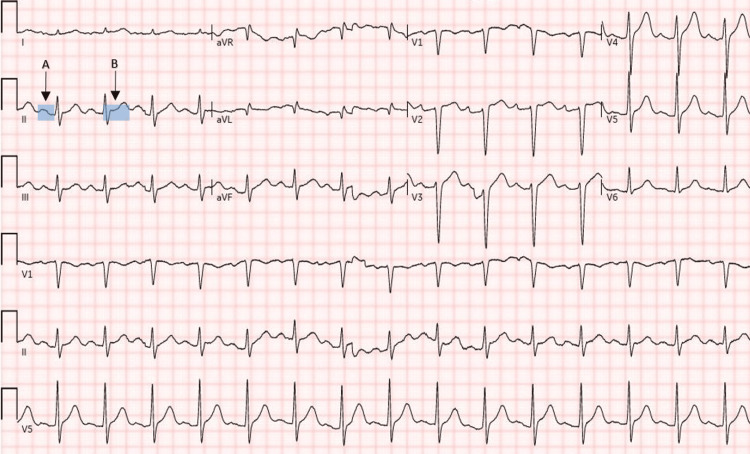
An ECG demonstrated sinus rhythm with a prolonged PR interval (arrow A with highlighted area) and a prolonged QTc interval (arrow B with highlighted area)

The initial ECG demonstrated sinus rhythm at 98 beats/min, first-degree atrioventricular block with a PR interval of 212 ms, and a prolonged corrected QT (QTc) interval of 490 ms (Figure 1). No acute ST-segment elevation, ST-segment depression, or T-wave abnormalities suggestive of acute myocardial ischaemia were identified.

Initial laboratory investigations performed at approximately 4:00 pm, around six hours after the collapse, demonstrated elevated cardiac troponin T levels, mild transaminitis, elevated glycated haemoglobin, increased total cholesterol levels, and a mildly elevated urea concentration (Table 1).

**Table 1:** Initial laboratory investigations performed approximately six hours after collapse eGFR (CKD-EPI): estimated glomerular filtration rate (chronic kidney disease-epidemiology collaboration); HbA1c: glycated haemoglobin; HDL: high-density lipoprotein; LDL: low-density lipoprotein.

Investigation	Results	Reference range
Cardiac troponin T	98 ng/L	0-13 ng/L
White blood cell count	5.28 × 10⁹/L	3.0-10.0 × 10⁹/L
Haemoglobin	127 g/L	130-170 g/L
Platelets	227 × 10⁹/L	150-400 × 10⁹/L
Urea	8.9 mmol/L	2.5-7.8 mmol/L
Creatinine	73 µmol/L	59-104 µmol/L
eGFR (CKD-EPI)	>90 mL/min/1.73 m²	—
Sodium	139 mmol/L	133-146 mmol/L
Potassium	3.7 mmol/L	3.5-5.3 mmol/L
Total cholesterol	5.5 mmol/L	0.0-4.9 mmol/L
Triglycerides	0.8 mmol/L	0.0-2.2 mmol/L
HDL cholesterol	2.7 mmol/L	0.5-3.5 mmol/L
Non-HDL cholesterol	2.8 mmol/L	0.0-3.9 mmol/L
LDL cholesterol	2.5 mmol/L	—
Cholesterol:HDL ratio	2.0	0.0-3.9
HbA1c	159 mmol/mol	20-41 mmol/mol
Total bilirubin	8 µmol/L	0-20 µmol/L
Alanine aminotransferase	52 U/L	10-50 U/L
Alkaline phosphatase	99 U/L	30-130 U/L
Total protein	48 g/L	60-80 g/L
Albumin	29 g/L	35-50 g/L
Creatine kinase	307 U/L	39-308 U/L
Aspartate aminotransferase	59 U/L	10-50 U/L

A repeat troponin T measurement performed four hours later increased to 105 ng/L.

Given the unwitnessed collapse, computed tomography of the head, cervical spine, thorax, abdomen, and pelvis was performed and demonstrated no acute intracranial pathology, fractures, or significant thoracoabdominal abnormalities.

The patient was initially managed as a case of heat stroke. His temperature improved with active cooling and intravenous fluid therapy, accompanied by the recovery of neurological function. However, due to the elevated troponin levels, cardiovascular risk factors, and unexplained collapse, the case was discussed with the cardiology team.

The cardiology team felt that non-ST-elevation ACS could not be excluded. It was considered that ACS may have precipitated the collapse or may have been triggered by the physiological stress associated with heat stroke. Consequently, he was commenced on dual antiplatelet therapy and standard ACS treatment.

Following recovery, a detailed history was obtained. The patient denied chest pain, palpitations, dizziness, or presyncopal symptoms before, during, or after the event. Troponin T, measured 29 hours after the collapse, had decreased to 75 ng/L. He remained free of chest pain throughout his admission, and a repeat ECG performed after 24 hours showed no ischaemic changes. Continuous cardiac monitoring demonstrated resolution of the initially prolonged PR interval and QTc, with no clinically significant arrhythmias detected.

Transthoracic echocardiography was performed approximately 24 hours after admission and demonstrated hypokinesia of the apical anterior, apical anteroseptal, and apical inferoseptal walls, with an overall left ventricular ejection fraction of 51%.

**Figure 2:**
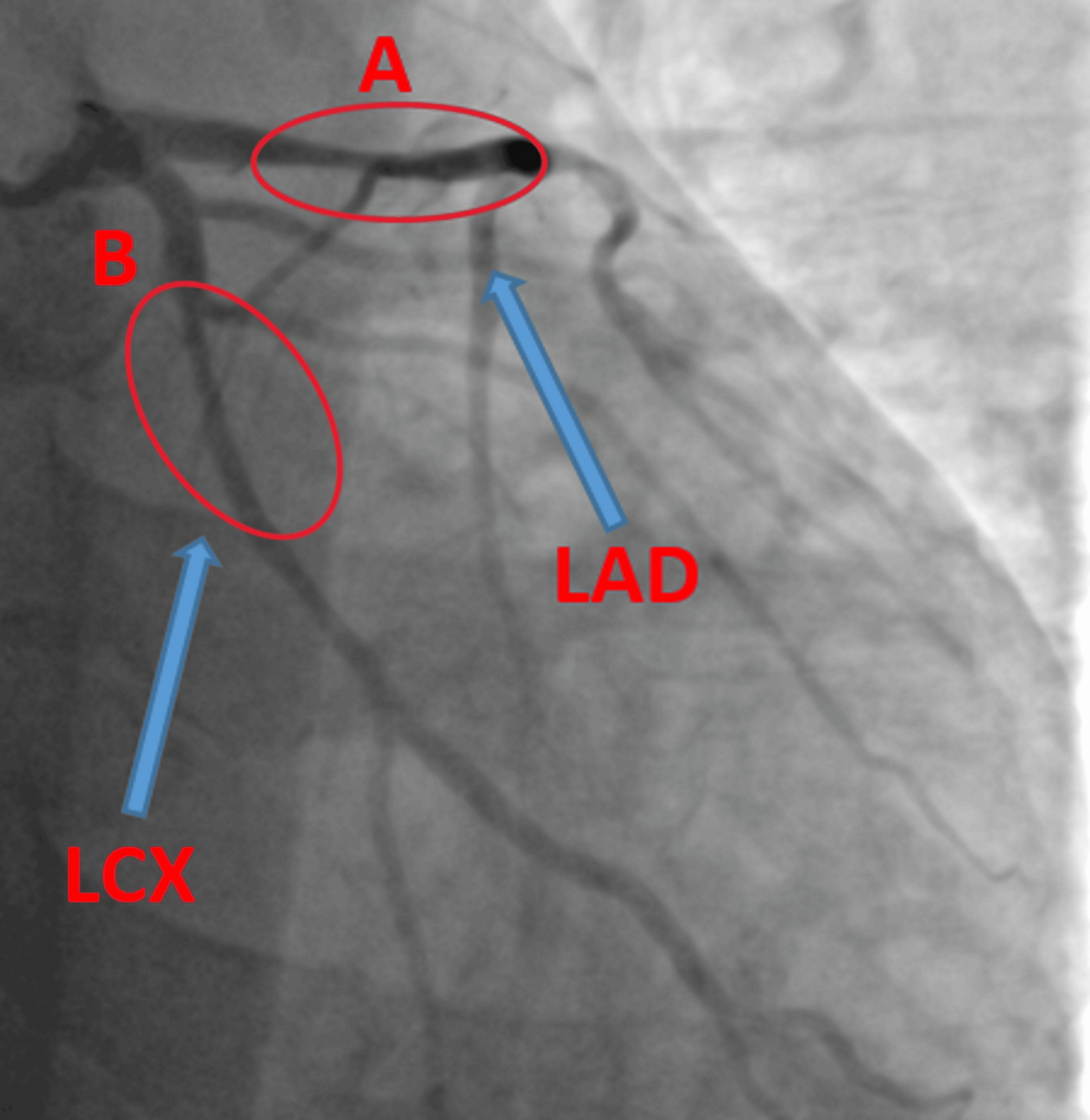
Coronary angiogram demonstrated moderate-to-severe (75%–94%) stenosis of the proximal LAD artery (circle A) and moderate stenosis of the proximal LCX artery (circle B). The right arrow indicates the course of the LAD artery, and the left arrow indicates the course of the LCX artery. LAD: left anterior descending; LCX: left circumflex.

**Figure 3:**
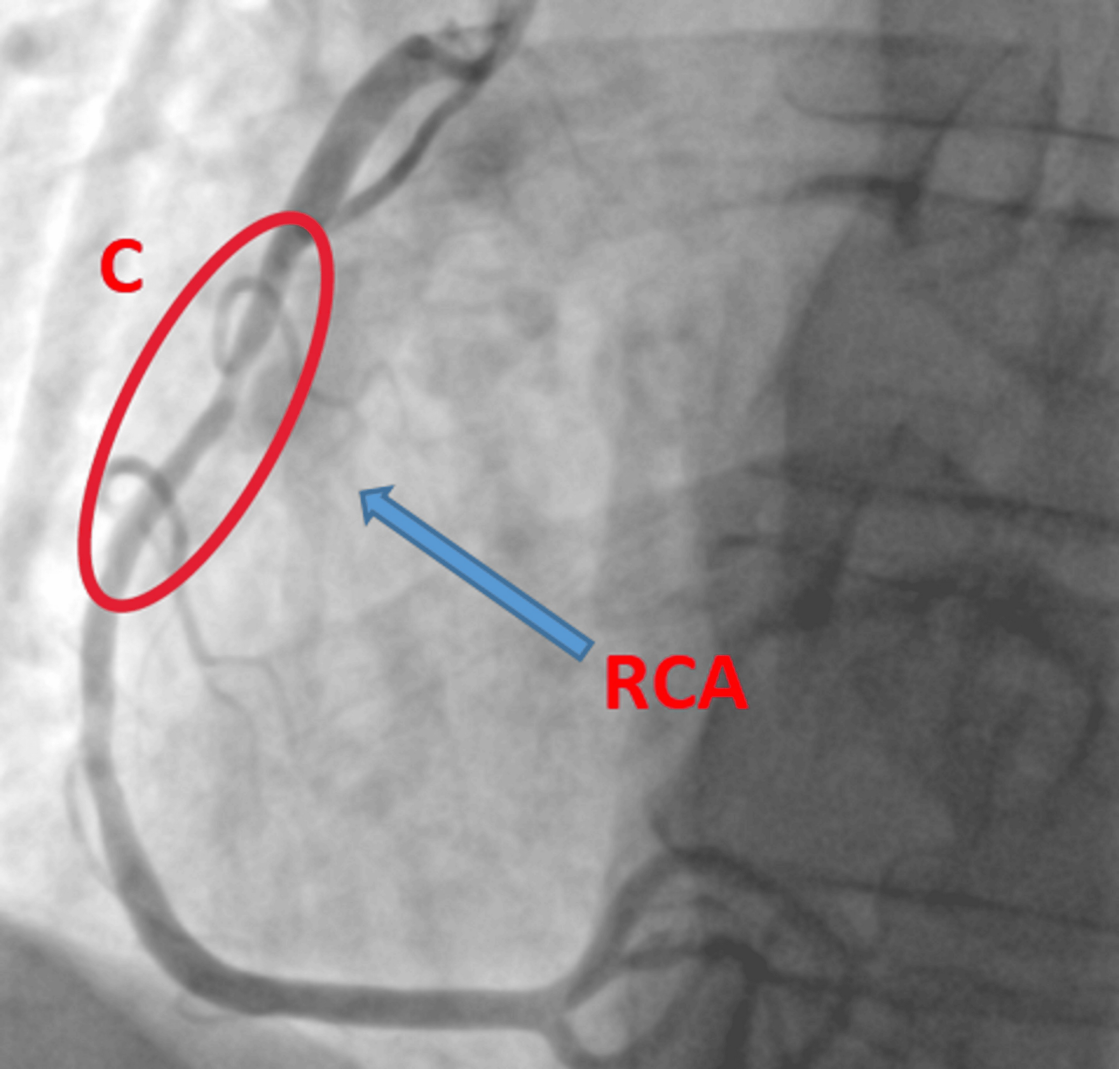
Coronary angiogram demonstrated moderate mid-vessel stenosis in the RCA (circle C). The arrow indicates the course of RCA. RCA: right coronary artery.

Given the combination of syncope, elevated troponin levels, regional wall motion abnormalities, and multiple cardiovascular risk factors, invasive coronary angiography was undertaken during the same admission. This demonstrated moderate-to-severe (75%-94%) stenosis of the proximal left anterior descending (LAD) artery, moderate proximal left circumflex artery disease, and moderate mid-vessel disease in a dominant right coronary artery (Figures 2, 3).

PCI with stenting of the proximal LAD was successfully performed during the same admission, as fractional flow reserve assessment confirmed that the coronary stenosis was haemodynamically significant. No evidence of intracoronary thrombus was identified. A staged PCI to the right coronary artery and left circumflex artery was planned following discharge. The patient was discharged on dual antiplatelet therapy and guideline-directed secondary prevention for coronary artery disease. He was also reviewed by the diabetes and endocrinology team because of markedly uncontrolled diabetes mellitus, and his glucose-lowering therapy was optimised to reduce future cardiovascular risk.

Written informed consent was obtained from the patient for publication of this case report and accompanying images.

## Discussion

This case highlights the diagnostic challenge of distinguishing heat stroke-related myocardial injury from ACS. The patient presented with classical features of heat stroke, including profound hyperthermia, altered consciousness, and rapid neurological improvement following active cooling. However, high-sensitivity troponin levels demonstrated a dynamic pattern (98 → 105 → 75 ng/L), with values exceeding the 99th percentile URL and exhibiting a subsequent rise and fall, consistent with acute myocardial injury according to the Fourth UDMI [6]. Elevated troponin levels, regional wall motion abnormalities on echocardiography, and significant cardiovascular risk factors prompted further cardiac investigation.

Cardiac involvement is increasingly recognised in heat stroke and encompasses a broad spectrum of manifestations, including myocardial injury, arrhythmias, conduction abnormalities, ventricular dysfunction, and heart failure [1,2]. Elevated troponin concentrations are frequently observed and may occur in the absence of obstructive coronary artery disease, reflecting direct myocardial injury secondary to hyperthermia, systemic inflammation, endothelial dysfunction, and microvascular impairment [2,3]. Furthermore, elevated biomarkers of myocardial stress, including natriuretic peptides, have been associated with more severe disease and adverse outcomes [5].

The initial electrocardiogram in our patient demonstrated first-degree atrioventricular block and QTc prolongation without acute ischaemic changes. Such conduction and repolarisation abnormalities have previously been described in heat stroke and may result from transient myocardial involvement, autonomic dysfunction, electrolyte disturbances, or direct thermal injury [1,2].

Heat exposure may also precipitate genuine ACS. Increased myocardial oxygen demand, dehydration, haemoconcentration, reduced coronary perfusion, and activation of inflammatory and prothrombotic pathways may collectively contribute to plaque instability and myocardial ischaemia in susceptible individuals [4]. Therefore, elevated troponin concentrations in patients with heat stroke should not automatically be attributed to heat-related myocardial injury alone.

An important limitation of this case is the uncertainty regarding the precise mechanism underlying the observed myocardial injury. Although coronary angiography demonstrated significant proximal LAD artery disease requiring revascularisation, it remains difficult to determine whether the presentation represented a Type 1 non-ST-elevation MI caused by plaque instability, a Type 2 MI secondary to the physiological stress of severe heat stroke, or myocardial injury occurring in the presence of incidental but clinically significant coronary artery disease. Contemporary literature increasingly recognises the challenge of differentiating MI from non-ischaemic myocardial injury in patients presenting with elevated cardiac biomarkers and complex physiological stress states [7]. Similar diagnostic uncertainty has been reported in recent cases of heat stroke associated with marked troponin elevation and variable angiographic findings ranging from angiographically normal coronary arteries to significant obstructive coronary artery disease [2,3].

Despite this uncertainty, the presence of regional wall motion abnormalities on echocardiography together with significant obstructive coronary artery disease on coronary angiography justified invasive assessment and revascularisation. This case emphasises the importance of maintaining a broad differential diagnosis and considering further cardiac investigation in patients with heat stroke who demonstrate persistent biomarker elevation, significant cardiovascular risk factors, unexplained syncope, or abnormal cardiac imaging findings.

## Conclusions

Heat stroke can cause elevated troponin levels, electrocardiographic abnormalities, and myocardial dysfunction that closely resemble ACS. However, these findings should not automatically be attributed to heat-related myocardial injury alone.

This case demonstrates that significant obstructive coronary artery disease may coexist with heat stroke and may only become apparent after comprehensive cardiac investigation. Patients presenting with heat stroke and evidence of myocardial injury should undergo careful cardiovascular assessment, particularly when cardiovascular risk factors, unexplained syncope, or abnormal cardiac imaging findings are present.
